# Improved Strength-Ductility of Ti-6Al-4V Casting Alloys with Trace Addition of TiC-TiB_2_ Nanoparticles

**DOI:** 10.3390/nano10122330

**Published:** 2020-11-24

**Authors:** Yunlong Zhu, Qinglong Zhao, Xiao Liu, Run Geng, Bao Wang, Qichuan Jiang

**Affiliations:** 1 State Key Laboratory of Automotive Simulation and Control, Jilin University, Changchun 130025, China; yunlong18@mails.jlu.edu.cn (Y.Z.); xiaoliu19@mails.jlu.edu.cn (X.L.); gengrun18@mails.jlu.edu.cn (R.G.); wangbao18@mails.jlu.edu.cn (B.W.); 2 Key Laboratory of Automobile Materials, Ministry of Education and School of Materials Science and Engineering, Jilin University, No. 5988 Renmin Street, Changchun 130025, China

**Keywords:** nanoparticles, nano-alloy, casting, titanium alloys, mechanical properties, grain refining

## Abstract

In this work, a high strength–ductility Ti64 cast alloy, containing trace TiC-TiB_2_ nanoparticles, was fabricated by adding dual-phased nano-TiC-TiB_2_/Al master alloys to the molten Ti64 alloys. The trace addition of the TiC-TiB_2_ nanoparticles (0.1 wt%) simultaneously reduced the size of the β grains, the α laths, and the α colony size of the lamellar structure during casting and suppressed the coarsening of the α laths during heat treatment. The yield strength and the uniform elongation of TiC-TiB_2_/Ti64 were increased by ~130 MPa and 2%, respectively. The simultaneously improved strength and ductility of the TiC-TiB_2_/Ti64 were attributed to the decrease in the α colony size of the lamellar structure, the significant refinement of the grains and α laths, and the pinning effect of nanoparticles.

## 1. Introduction

Titanium alloys exhibit high specific strength and stiffness, excellent corrosion resistance, and good biocompatibility, and they have been widely used in many fields, such as aerospace [1], automobile [2], biomedicine [3], and offshore industries. The most extensively used titanium alloy is Ti-6Al-4V (Ti64), accounting for roughly half of the total usage of commercial titanium alloys [4]. Ti-6Al-4V is a typical α + β titanium alloy, which can obtain various microstructures through different heat treatments, thus achieving diverse mechanical properties [5].

Over the past few decades, ceramic particles such as TiC [6,7], TiB [8], SiC [9,10], ZrN [11], TiN [12] have been used to reinforce titanium alloys. Among these particles, TiC and TiB [12,13,14,15,16,17,18] are widely used as reinforcements in the composites because of their high modulus, their excellent thermal stability, their similar density with titanium, their low lattice mismatch and thermal expansion coefficient with titanium, and their clean interface without any unfavorable reaction between particles and the titanium matrix. Many studies have confirmed that the grain and the α-Ti lath colony of the titanium alloy were refined, and the mechanical properties were improved by adding TiB, TiC, or both [16,19,20,21]. Patil et al. [22] prepared TiB-reinforced Ti64 alloys with direct metal laser sintering and concluded that the martensitic laths were effectively refined with the addition of TiB and that the hardness and wear performance were improved. H.K.S. Rahoma et al. [19] prepared titanium alloy reinforced with 2.2 vol% micron-sized TiC-TiB particles, indicating that the strength was improved and that the grains were obviously refined. Ti alloys reinforced by micron-sized TiC-TiB particles have been reported in many articles [19,20,21]. The reinforced particles reported in the literature are mostly micron-sized [12,13,14,15,16,17,18,19,20,21]. Nano-sized reinforced particles have been proven to have a stronger strengthening effect than micron-sized particles in the powder metallurgy of Ti-base composites [23]. Recent studies on additive manufacturing of Ti alloys reported that nano-sized particles not only refined the grains and the α-Ti lath colony but also increased the mechanical properties by pinning dislocation [24,25,26].

Researchers fabricated ceramic particle-reinforced titanium alloy by Spark Plasma Sintering (SPS), additive manufacturing, forging and rolling, and hot isostatic pressing. The high cost of thermomechanical processing or additive manufacturing has stimulated the continuous effort to develop and improve the casting methods for titanium alloys [27]. Investment-cast titanium alloys, which can meet the stringent requirements for structural components and better utilize available metal or reduce the cost of machining or forming complex parts, are increasingly being recognized as affordable solutions. The mechanical properties of cast Ti alloys are lower than those of the Ti alloys prepared by forging and rolling, powder metallurgy, and hot isostatic pressing. The enhancement of the cast titanium alloys via the addition of nanoparticles is a promising method, which can ensure the dimensional accuracy of the workpiece. However, the influence of nanoparticles on cast titanium alloys has been rarely reported.

In summary, extensive works have been conducted on micron and high weight ratio particle-reinforced Ti64, but few have examined trace nanoparticle-reinforced cast Ti64. In this study, the TiC-TiB_2_/Ti64 was fabricated by adding dual-phased TiC-TiB_2_/Al master alloys to the molten Ti64 alloys. The effects of dual-phased nanoparticles (0.1 wt%) on the microstructure and tensile properties of the TiC-TiB_2_/Ti64 were investigated, and the related mechanisms were discussed. This study provides a novel method for improving the mechanical properties of Ti cast alloys for industrial production.

## 2. Materials and Methods

The raw Ti64 (Ti-6.1Al-4.0V, in wt%) alloy billets were provided by Western Superconducting Technologies Co. Ltd. (Xi’an, China). The 0.1 wt% TiC-TiB_2_/Ti64 alloy was prepared by adding nano-TiC-TiB_2_/Al master alloys to the melted Ti64 alloy at 1973 K in a vacuum induction furnace. The melt was held for three minutes and then poured into a graphite mold. The 30 wt% nano-TiB_2_-TiC/Al alloy was prepared by combustion synthesis of 70Al-21.7Ti-8.3nano-B_4_C powder system (in wt%), which has been described in [28]. The molar ratio of TiB_2_ to TiC was 2:1. The average diameters of TiC and TiB_2_ were 88 and 280 nm, respectively (Figure S1). The heat treatment (duplex anneal) involved solution treatment (S) at 1193 K for one hour in a vacuum, then air cooling and aging (A) at 823 K for four hours.

The microstructure was characterized using the optical microscope (OM), a scanning electron microscope (SEM, vega3 XMU Tescan, CZ), and a transmission electron microscope (TEM, JEM-2100F JEOL, Japan). The specimens for SEM and OM observation were polished and etched in Keller’s reagent (3 mL HF, 6 mL HNO_3_, 91 mL H_2_O) for 30 s at room temperature. The specimens for TEM observation were ground to the thickness of 25~30 μm and then thinned by an ion beam thinner (RES101). Tensile samples with a cross-section of 4.5 × 1.5 mm and a gauge length of 10 mm were used for the tensile test. The tensile tests were carried out at room temperature using the MTS-810 testing machine at a constant ramp speed of 0.5 mm/min. Each measurement was repeated four times for each alloy.

## 3. Results

**Figure 1:**
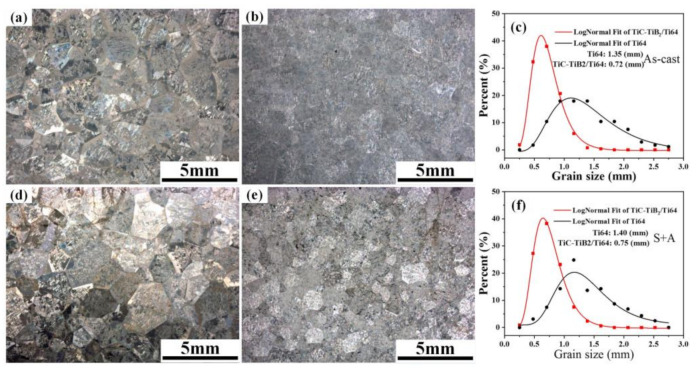
Metallography of Ti64 and TiC-TiB_2_/Ti64. (**a**) As-cast Ti64. (**b**) TiC-TiB_2_/Ti64. (**c**) The statistical results of the grain size of as-cast samples. (**d**) Ti64 after heat treatment. (**e**) TiC-TiB_2_/Ti64 after heat treatment. (**f**) The statistical results of the grain size after heat treatment.

There is no apparent change in the grain size of as-cast samples and the samples after heat treatment, as shown in Figure 1a,b,d,e. The average grain size of TiC-TiB_2_/Ti64 was smaller than that of Ti64. The grain refinement of Ti64 by adding trace nano-TiC-TiB_2_ particles was significant (Figure 1c,f).

**Figure 2:**
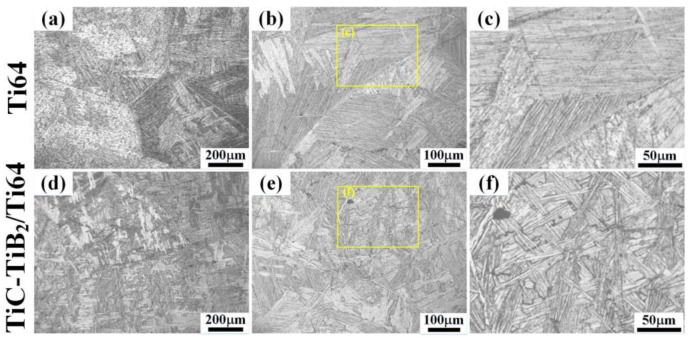
(**a**) Metallography of Ti64. (**b**,**c**) Metallographies of Ti64 after heat treatment. (**d**) Metallographies of TiC-TiB_2_/Ti64. (**e**,**f**) Metallographies of TiC-TiB_2_/Ti64 after heat treatment.

**Figure 3:**
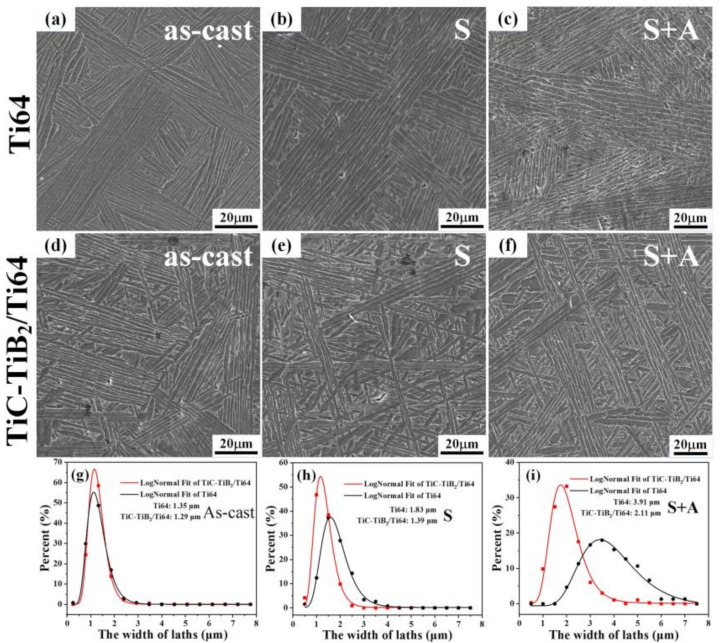
Microstructure analysis of the samples. (**a**–**c**) SEM images of Ti64. (**d**–**f**) SEM images of TiC-TiB_2_/Ti64. (**g**–**i**) The corresponding statistical results of the width of the laths (S: solution treatment; S+A: solution and aging treatment).

**Figure 4:**
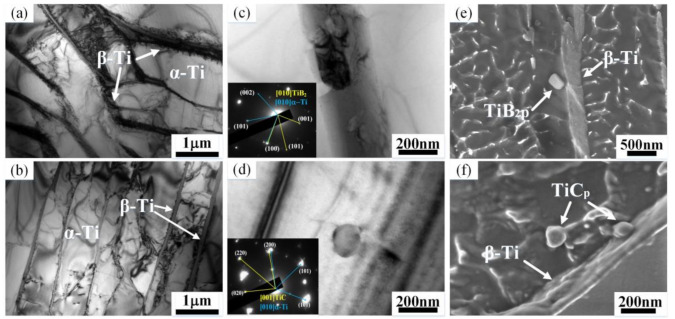
TEM images analysis of Ti64 and TiC-TiB_2_/Ti64 after heat treatment. (**a**,**b**) TEM images of Ti64 and TiC-TiB_2_/Ti64. (**c**,**d**) TEM images of TiB_2_ and TiC particles in TiC-TiB_2_/Ti64 and their SAED patterns. (**e**,**f**) FESEM images of TiC and TiB_2_.

After heat treatment, the size of α colony did not change significantly, as shown in Figure 2a,b,d,e. The α laths of the as-cast TiC-TiB_2_/Ti64 show a basket weave structure in Figure 2d–f. The α colony size of the lamellar structure in Ti64 is larger than that in TiC-TiB_2_/Ti64 (Figure 2c,f). After heat treatment, the width of the laths in Ti64 (3.91 μm) is wider than that in the TiC-TiB_2_/Ti64 (2.11 μm), as show in Figure 3c,f,i. Figure 3 suggests that adding trace nano-TiC-TiB_2_ particles to the Ti64 alloys can effectually retard the coarsening of the α laths during heat treatment. The widths of α-Ti and β-Ti in TiC-TiB_2_/Ti64 are thinner than those in Ti64 (Figure 4a,b). Figure 4c–f shows that the particles are located in the α-Ti, close to the boundary of α-Ti/β-Ti. Some particles are located inside the α-Ti laths, as shown in Figures S2 and S3. The (200) crystal plane of the TiC is parallel to the (200) crystal plane of α-Ti (Figure 4c). The (100) crystal plane of TiB_2_ is parallel to the (100) crystal plane of α-Ti (Figure 4d).

**Figure 5:**
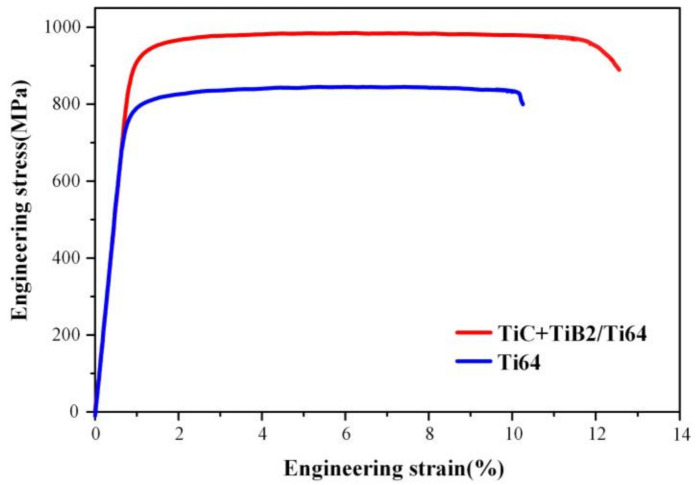
The engineering tensile stress–strain curves of Ti64 and TiC-TiB_2_/Ti64 after heat treatment.

The engineering tensile stress–strain curves of Ti64 and TiC-TiB_2_/Ti64 after heat treatment (Figure 5) suggest that the TiC-TiB_2_ nanoparticles improve the tensile properties; the yield strength, the ultimate strength, and the uniform elongation of TiC-TiB_2_/Ti64 are increased by 130.0 MPa, 130.6 MPa, and 2.0%, respectively. We make present the details of the tensile tests in Figure S4 and Table S1.

## 4. Discussion

In the present work, TiB_2_ existed instead of TiB, which was thermodynamically favored in Ti alloys containing trace addition of B. The theoretical value of B content in TiC-TiB_2_/Ti64 is 0.026 wt%, which was calculated based on the nominal amount of TiB_2_. According to the Ti-B phase diagram [29], this value is within the maximum solubility of B in the Ti matrix. When the holding time is long enough, the TiB_2_ particles are expected to dissolve into the Ti matrix at high temperatures. We found TiB_2_ particles in the Ti64 matrix, as shown in Figure 4c,e. Thus, we believe that the undissolved TiB_2_ particles in the TiC-TiB_2_/Ti64 may be due to the short holding time prior to casting.

The addition of TiC-TiB_2_ induced grain refinement in Ti64 (Figure 1). Table 1 shows that the Bramfitt two-dimensional lattice mismatch between particles and β-Ti is less than 12%, indicating that the TiC and TiB_2_ particles could act as heterogeneous nucleation sites for the β-Ti grains. The TiC/TiB_2_ nanoparticles in front of the solid–liquid interface might hinder the growth of β-Ti grain during solidification [30]. The grain of Ti64 is refined by adding TiC-TiB_2_ nanoparticles. The V solutes increase the stability of β-Ti. The enrichment of the V element adjacent to the β-Ti grain boundaries is ascribed to the solute partition during non-equilibrium solidification (Figure S5). Gil et al. found that the Al content gradually decreases from the center of the α lath to the β phase, while the V content increases along the same direction in the as-cast Ti64 [31]. The α phase is first precipitated along the β grain boundaries, rather than inside the grains [32]. The heat treatment induced homogenization and reduced the V content at the grain boundaries, promoting the nucleation of α-Ti during the β-Ti → α-Ti phase transformation at the grain boundaries. The nanoparticles refined the grains. Therefore, the nucleations of α-Ti increase; thus, the size of the α-Ti colony and the width of the α-Ti lath decrease.

Shibayan Roy found that the oxygen content around the TiB particles is higher than the oxygen content at the grain boundaries due to the TiB interiors from the β grain boundaries having a high propensity of grain boundary diffusion [33]. Z. Q. Chen proved that Ti_2_C particles have a similar effect [34]. Hence, we speculate that TiC and TiB_2_ nanoparticles also imbibe oxygen from the β grain boundaries. O element is a strong stabilizer of α-Ti. In addition, the lattice mismatch between TiC/TiB_2_ and α-Ti is less than 12% (Table 1). Therefore, the α-Ti nucleates at the location of particles with high oxygen content. The nucleation sites of the α-Ti increase. The size of the α-Ti colony and the width of the α lath decrease. Besides, TiC-TiB_2_ particles might hinder the movement of α-Ti boundaries. The coarsening of the α laths of the TiC-TiB_2_/Ti64 was retarded compared to that of Ti64 during heat treatment.

The mechanical properties of Ti64 with a fully lamellar microstructure are determined by the α colony size of the lamellar structure and the size of the α laths [35]. The simultaneously increased strength and ductility (Figure 4), by adding TiC-TiB_2_ particles, are attributed to the decrease in the α colony size of the lamellar structure (the size of the α colony, as shown in Figure S6), the refinement of the α laths, and the pinning effect of nanoparticles on dislocations in the matrix. It has been suggested that the α/β interfaces are similar to grain boundaries and that the Hall–Petch strengthening depends on the α lamellar thickness [36]. According to the classic Hall–Petch equation, the strength increment is expressed as:

(1) $$\Delta \sigma_{H-P}{=K}_{y}\left({d_{y}}^{-0.5}-d_{0}{}^{-0.5}\right)$$

where Δ*σ_H-P_* is the yield strength increment of TiC-TiB_2_/Ti64 as compared to Ti64 after heat treatment because of the refinement of the martensitic laths; *K*_y_ is the strengthening coefficient with a value of 320 MPa·μm^1/2^ [37,38]. *d_y_* and *d_0_* are the average widths of the α laths in the TiC-TiB_2_/Ti64 and Ti64 after heat treatment, respectively. The calculated yield strength increment is 58.4 MPa.

Jiang et al. [39] have reported that micron-sized (>1 μm) particles have a significant effect on the load-bearing strengthening of the composites, while nano-sized (<0.1 μm) and submicron-sized (0.1–1 μm) particles increase the strength mainly via the Orowan mechanism. The strengthening of nano-TiC-TiB_2_ particles can be calculated by the Orowan–Ashby equation as follows:

(2) $$\Delta \sigma_{O-A}=0.81MGb{\left(2\pi \lambda \right)}^{-1}\cdot \ln r/b$$

where ∆*σ_O-A_* is the strengthening effect caused by Orowan stress of particles; *M* is the Taylor factor, for Ti64 alloy with a value of 3.06 [40]; *b* is the Burgers vector of the matrix (0.295 nm) [41]; *G* is the shear modulus of Ti64 matrix, with a value of 46 GPa [42]; *r* is the radius of particles; *λ* can be calculated as follows:

(3) $$\lambda =0.4d[(\pi /f_{v}{)}^{0.5}-2]$$

where *f_v_* and *d* are the volume fraction and diameter of particles. The calculated yield strength increment is around 16.9 MPa. The α colony size of the lamellar structure also strongly affects the strength, but the contribution could not be estimated due to the lack of relevant data. The increased strength in TiC-TiB_2_/Ti64 is mainly attributed to the refinement of the α-laths, the α colony size of the lamellar structure, and the Orowan strengthening effect of nanoparticles.

## 5. Conclusions

Adding trace TiC-TiB_2_ nanoparticles (0.1 wt%) via TiC-TiB_2_/Al master alloys to Ti64 melt during casting resulted in grain refinement (by ~50%). The TiC-TiB_2_ particles might have acted as nucleation sites for β-Ti and hindered the movement of the β-Ti grain boundary. The TiC-TiB_2_ nanoparticles decreased the width of the α laths and the size of the α colony, which was facilitated by the increased nucleation sites provided by the increase in grain boundaries and TiC-TiB_2_ nanoparticles. The TiC-TiB_2_ nanoparticles also suppressed the coarsening of the α laths during heat treatment. The yield strength and the uniform elongation of Ti64 alloys were increased by TiC-TiB_2_ from 793.3 to 923.6 MPa and from 9.2 to 11.1%, respectively. This study provides a novel method for strengthening cast titanium alloys which is potentially applicable in the industrial manufacturing of particle-strengthened cast titanium alloys.

## Figures and Tables

**Table 1:** The two-dimensional lattice mismatch between TiC-TiB_2_ particles and Ti matrix.

Orientation	(100)_TiB2_//(100) _β-Ti_	(200)_TiC_//(100) _β-Ti_	(100)_TiC_//(110) _β-Ti_	(200)_TiC_//(200) _α-Ti_	(001)_TiB2_//(001) _α-Ti_
Mismatch	5.83%	1.57%	9.3%	7.06%	2.6%

## Supplementary Materials

The following are available online at https://www.mdpi.com/2079-4991/10/12/2330/s1, Figure S1. The FESEM images of TiC-TiB_2_ particles (a,b) and the statistical results of the diameter of the TiC and TiB_2_ (c,d), Figure S2. TEM images of TiC and TiB_2_ particles in TiC-TiB_2_/Ti64 (a,b); FESEM patterns of TiC-TiB_2_/Ti64 (c,d), Figure S3. TEM and FESEM images of TiC and TiB_2_ in TiC-TiB_2_/Ti64. a, b: the location of TiB2 particles; c-f: the locations of TiC particles, Figure S4. Engineering strength-Strain curves, Figure S5. (a) The content changes of Al and V element in β-Ti during solidification. (b) The content changes of Al and V element in β-Ti when the temperature is below the phase transform point, Figure S6. The statistical results of the size of α-Ti colony, Table S1. Details of the tensile tests performed on the cast Ti64 and TiC-TiB_2_/Ti64 after heat treatment.
