# Supplementary material for: Improved Strength-Ductility of Ti-6Al-4V Casting Alloys with Trace Addition of TiC-TiB2 Nanoparticles
Source: Nanomaterials (Basel). 2020 Nov 24;10(12):2330. doi: 10.3390/nano10122330 (PMC7760530; doi:10.3390/nano10122330)
Supplement: Supplementary file 1 [file nanomaterials-10-02330-s001.pdf]

nanomaterials

MDPI

Supplementary Material

Improved Strength-Ductility of Ti-6Al-4V Casting Alloys with Trace Addition of $TiC-TiB_2$ Nanoparticles

Yunlong Zhu [1,2], Qinglong Zhao [1,2,*], Xiao Liu [1,2], Run Geng [1,2], Bao Wang [1,2] and Qichuan Jiang [1,2,*]

[1] State Key Laboratory of Automotive Simulation and Control, Jilin University, Changchun 130025, China; yunlong18@mails.jlu.edu.cn (Y.Z.); xiaoliu19@mails.jlu.edu.cn (X.L.); gengrun18@mails.jlu.edu.cn (R.G.); wangbao18@mails.jlu.edu.cn (B.W.)

[2] Key Laboratory of Automobile Materials, Ministry of Education and School of Materials Science and Engineering, Jilin University, No. 5988 Renmin Street, Changchun 130025, China

* Correspondence: zhaoqinglong@jlu.edu.cn (Q.Z.); jqc@jlu.edu.cn (Q.J.)

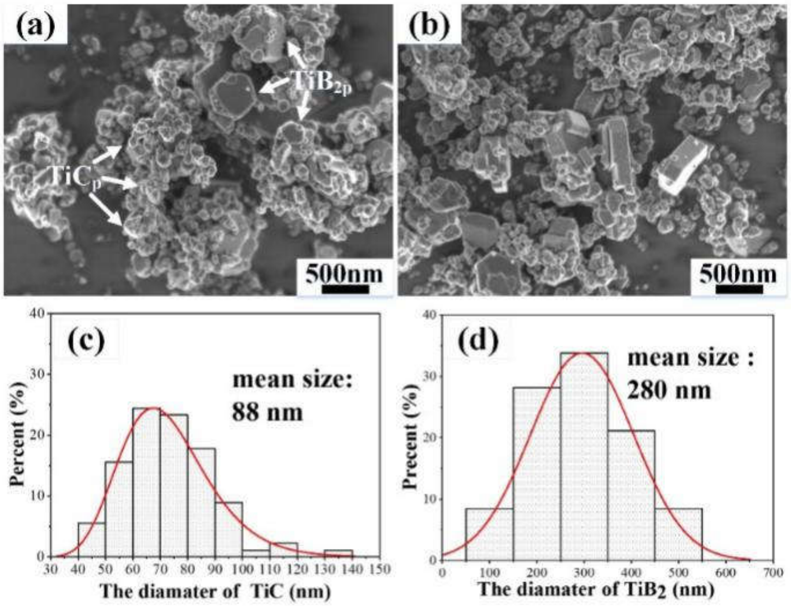

Figure S1. The FESEM images of $TiC-TiB_2$ particles (**a**–**b**) and the statistical results of the diameter of the TiC and TiB_2 (**c**–**b**).

The TiC and TiB_2 particles were obtained by extracting from the $TiC-TiB_2$/Al master alloys. The extractant was 90% hydrochloric acid. The size of spherical TiC particles is small. TiB_2 particles are regular hexagon or rectangle and large in size. The areal number density of TiC is more than that of TiB_2.

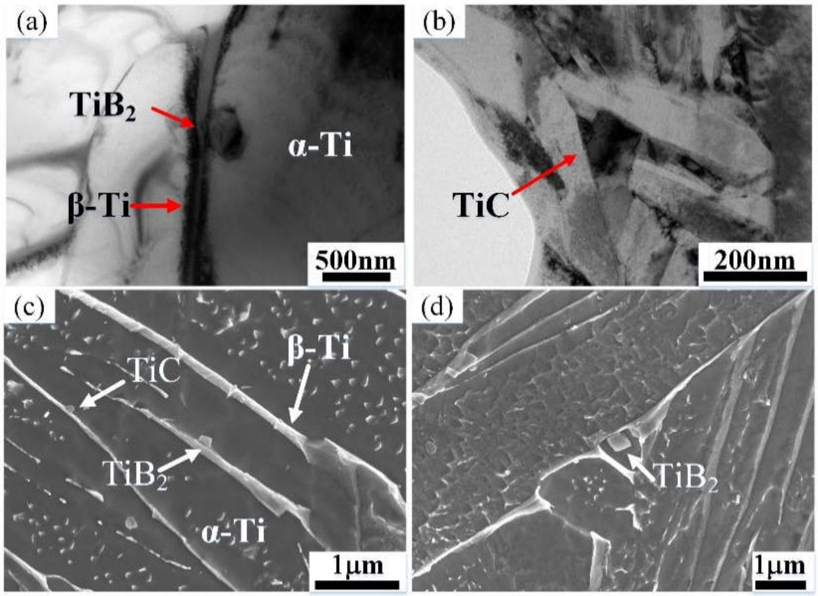

Figure S2. TEM images of TiC and TiB_2 particles in TiC-TiB_2/Ti64 (**a**,**b**); FESEM patterns of TiC-TiB_2/Ti64 (**c**,**d**).

Figure S2 shows that TiC and TiB_2 particles are located at the border of α-Ti and β-Ti. Those particles may suppress the movement of the boundaries of α-Ti/β-Ti.

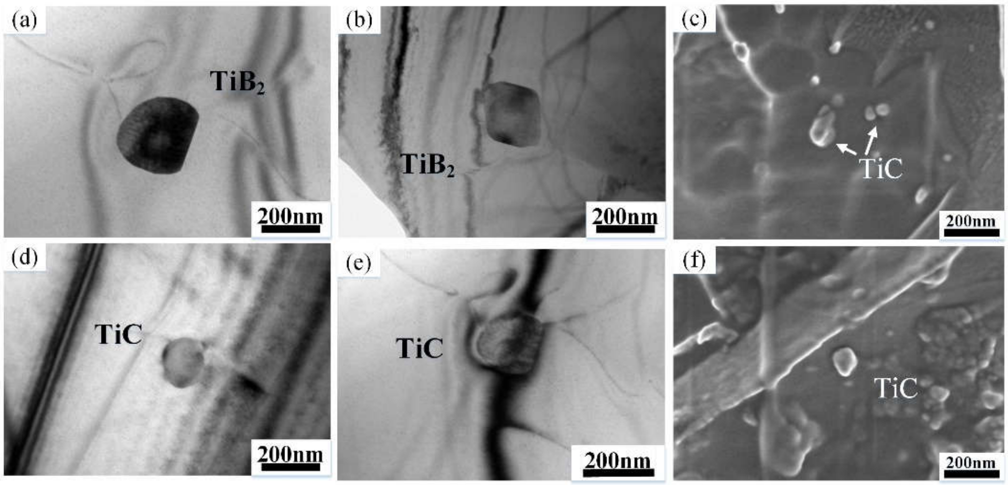

Figure S3. TEM and FESEM images of TiC and TiB_2 in TiC-TiB_2/Ti64. a, b: the location of TiB2 particles; c-f: the locations of TiC particles.

Figure S3 shows that some TiC, TiB_2 particles are inside the α laths, which may act as the nucleation site of α-Ti.

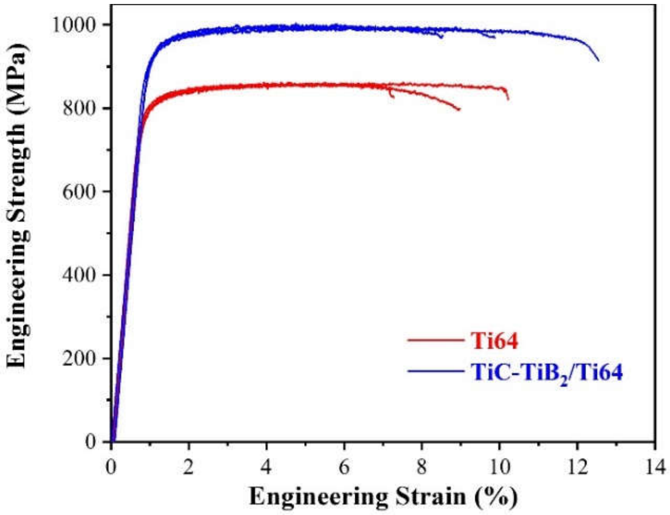

Figure S4. Engineering strength-Strain curves.

The data for the Ti64 and TiC-TiB_2/Ti64 after heat treatment tested in this study indicate good repeatability.

Table S1. Details of the tensile tests performed on the cast Ti64 and TiC-TiB_2/Ti64 after heat treatment.

Samples		$\sigma_{0.2}$ (MPa)	σ_{UTS} (MPa)	UE (%)
	Sample 1	926.2	992.6	11.2
TiC-TiB_2/Ti64	Sample 2	937.2	996.0	8.9
	Sample 3	925.2	990.7	7.8
	Sample 1	796.6	857.9	10.0
Ti64	Sample 2	794.1	859.0	7.4
	Sample 3	789.2	856.7	6.8

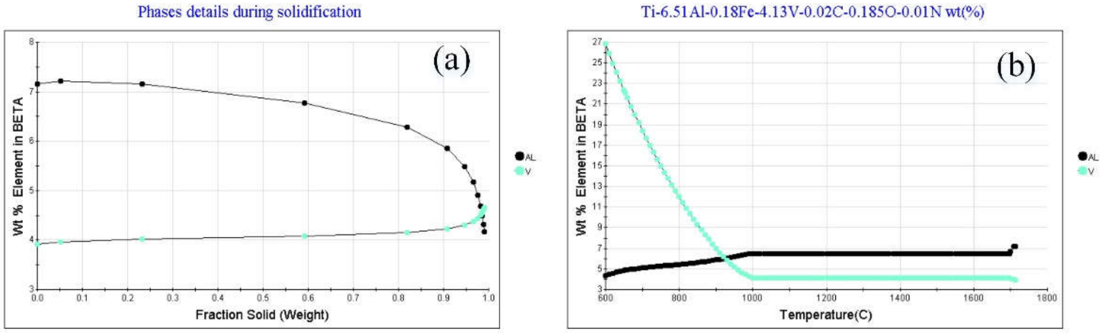

Figure S5. (**a**) The content changes of Al and V element in β-Ti during solidification. (**b**) The content changes of Al and V element in β-Ti when the temperature is below the phase transform point.

Figure S5 was calculated by JMatPro v10 (Sente Software Ltd.; 2001). Scheil calculations suggest that the element V accumulates at dendritic regions while Al is concentrated in the dendrite center of β-Ti (Figure S5a). The content of V element of β-Ti rises sharply, as the temperature decreases below the phase transition point, and the content of Al element of β-Ti is slightly reduced (Figure S5b).

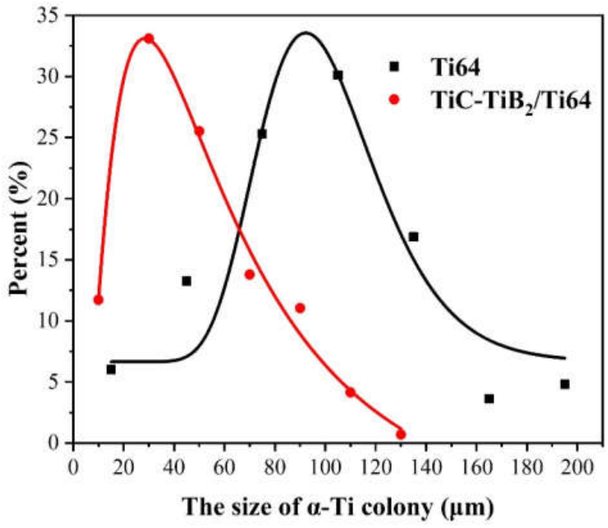

Figure S6. The statistical results of the size of α-Ti colony.

The mean size of α-Ti colony in Ti64 is 109 μm. The mean size of α-Ti colony in $TiC-TiB_2/Ti64$ is 43 μm. Ten metallographies are counted for each value.

Publisher's Note: MDPI stays neutral with regard to jurisdictional claims in published maps and institutional affiliations.

© 2020 by the authors. Licensee MDPI, Basel, Switzerland. This article is an open access article distributed under the terms and conditions of the Creative Commons Attribution (CC BY) license (http://creativecommons.org/licenses/by/4.0/).
